# Development of a pandemic-related core set of quality indicators for quality and patient safety in University Hospitals in Germany

**DOI:** 10.1186/s12913-024-12194-3

**Published:** 2025-01-08

**Authors:** Janis Evers, Natalie Altschuck, Claudia Mehl, Lilly Rüthrich, Lorenz Harst, Felix Walther, Oliver Steidle, Arnt Suckow, Ruth Hecker, Jochen Schmitt, Max Geraedts

**Affiliations:** 1 Institute for Health Services Research and Clinical Epidemiology, Faculty of Medicine, Philipps-University Marburg, Marburg, Germany; 2 Department of Health Sciences, University of Applied Sciences Fulda, Fulda, Germany; 3 Center for Evidence-Based Healthcare, Faculty of Medicine and University Hospital Carl Gustav Carus, Dresden University of Technology (TUD), Dresden, Germany; 4 Department of Quality and Clinical Risk Management, University Hospital Essen, Essen, Germany; 5 Department of Quality and Clinical Risk Management, University Medical Center, Georg-August-University Goettingen, Goettingen, Germany

**Keywords:** Pandemic, Quality indicators, University hospitals, Healthcare quality management, COVID-19, Patient safety

## Abstract

**Background:**

The COVID-19 pandemic entailed a global health crisis, significantly affecting medical service delivery in Germany as well as elsewhere. While intensive care capacities were overloaded by COVID cases, not only elective cases but also non-COVID cases requiring urgent treatment unexpectedly decreased, potentially leading to a deterioration in health outcomes. However, these developments were only uncovered retrospectively. Especially university hospitals, which were meant to take on a central coordinating role, did not have detailed information on expected healthcare utilization, available resources and capacities, and the quality of medical care.

The experience of compromised healthcare and a lack of monitoring during the COVID-19 pandemic made it clear that healthcare systems should be better prepared. Therefore, the aim of this work was to develop a set of indicators suited to detect undesirable developments concerning the provision of inpatient healthcare.

**Material & methods:**

The study employed a literature review, online surveys, expert interviews, and a multistep evaluation process to develop a core set of quality indicators (QIs) suitable for assessing the resilience of university hospitals during pandemics. This initial set of indicators was refined through consultations with a) quality and risk management officials from German university hospitals via an online survey and b) a diverse panel of experts.

**Results:**

The comprehensive evaluation identified two primary strands: organizational/management indicators (Strand A, 60 indicators) and disease-specific clinical quality and patient safety indicators (Strand B, 20 indicators for critical conditions like stroke, myocardial infarction, and cancer.) Three additional indicators were added after a final expert panel meeting, resulting in a final set of 83 indicators.

**Discussion and conclusion:**

The developed QIs mark a significant advancement in the operational preparedness of university hospitals for pandemics. The study contributes to quality management in healthcare during pandemics by creating the basis for a structured approach to pandemic preparedness and response. This unique set of QIs within the German context presents an opportunity for establishing quality improvement, underscoring the importance of a robust, adaptable quality management framework as a basis for safeguarding against future health crises.

**Supplementary Information:**

The online version contains supplementary material available at 10.1186/s12913-024-12194-3.

## Background

The COVID-19 pandemic has led to a significant decline in the utilization of medical services across Germany, notably affecting university hospitals [1, 2]. This decrease extended beyond elective procedures and also affected urgent non-COVID related medical cases, resulting in delayed admissions for acutely ill patients [3]. Simultaneously, the pandemic caused restrictions in intensive care capacities and therefore led to a disproportionate shortage in healthcare provision of chronically ill patients [4]. The steep increase in case numbers required a strategic restructuring of healthcare services, while the workforce faced increased levels of stress and absenteeism [5]. Additionally, rather generic frameworks commonly used in Germany and elsewhere up to this point, such as critical incidence reporting systems (CIRS) showed a decline in reporting during COVID waves, likely due to their lack of features specific to the pandemic and staff time constraints [6, 7]. These developments collectively impaired the quality and safety of patient care within German hospitals. At this critical juncture, university hospitals in Germany took a central coordinating role, marshaling their expertise to address the challenges posed by the pandemic [8, 9].

A primary focus of quality management within hospitals during this period was the development of a responsive framework to facilitate agile decision-making processes. The established hospital quality of care reporting system in Germany only covers about 20% of procedures and provides feedback with a time lag of about 2 years. Therefore, a quicker framework for continuous monitoring of inpatient health care provision was required to ensure continuity of medical care, protect healthcare workers, and adapt to the changing pandemic situation and associated policy decisions. The implementation of a monitoring system, guided by a solid set of quality indicators (QIs) and recommendations, could strengthen the healthcare system against future pandemics or similar crises and allows to maintain the quality and safety of university healthcare services.

Although there were proposals for pandemic-adapted indicators from some international governmental organizations (IGOs) [10–13], there was a need for a set that specifically addresses the German university medicine and covers the unique properties of the German healthcare system – such as specialty care in hospitals and in private practice settings, and a mixture of public, charity and private hospital care providers. Such, the coordinating role of university clinics in regional health care, which emerged during the pandemic, requires networking with both smaller hospitals within a certain hospital care cluster and with outpatient care providers. Additionally, an in-depth knowledge of regional infection dynamics and available health care resources is required – factors that were not measured routinely by university clinics at the onset of the pandemic [14–16]. The development of an indicator set was part of the PREPARED project (PREparedness and Pandemic Response in Germany), an initiative of the German Network University Medicine (NUM). The main objective of PREPARED is to establish a concept for a comprehensive, collaborative, adaptable and sustainable pandemic management and preparedness infrastructure that meets the above-mentioned need for a comprehensive pandemic preparedness framework and enables coordinated, prompt, accurate and evidence-based actions and responses to threats to patient care and public health in the event of a pandemic [9]. The effort presented here as a part of PREPARED sought to create a dual-phase concept for quality and risk management tailored to university hospitals, applicable during both pandemic (“crisis mode”) and inter-pandemic (“steady state”) periods.

## Material & methods

**Fig. 1:**
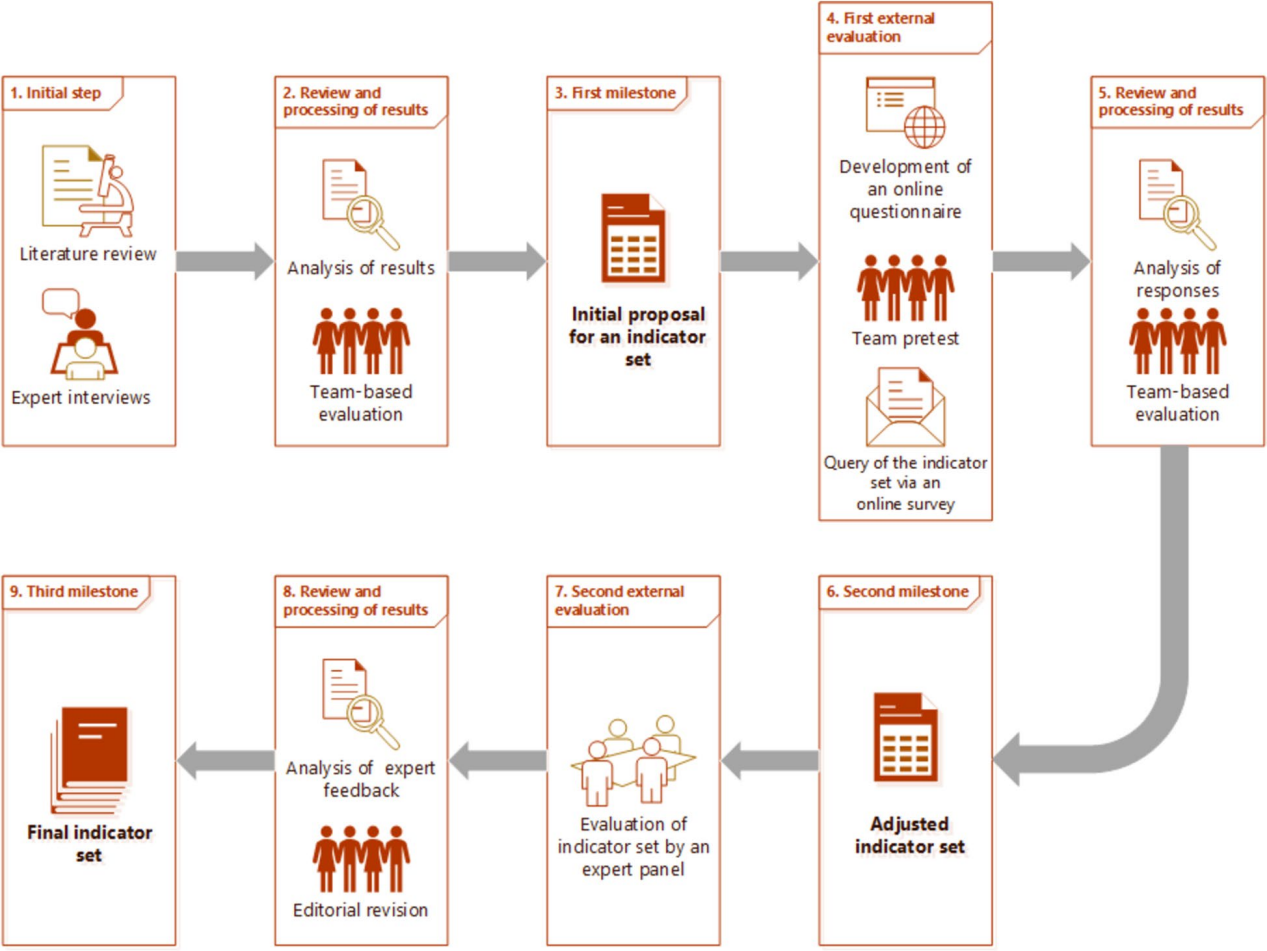
Overview of the complete workflow for the creation of the indicator set

The development of the core set for QIs was underpinned by an extensive literature review conducted across pertinent databases and IGOs, complemented by expert interviews with board members from the quality management (QM) and risk management (RM) departments of selected German university hospitals (step one). All steps taken to develop the final indicator set are depicted in Fig. 1.

The core QI-set development contained two primary strands:

Strand A aimed at identifying general indicators and recommendations pertaining to organizational and management practices from the realm of quality and risk management, encompassing both structural and process indicators. Strand B concentrated on disease-specific clinical quality and patient safety indicators and areas of treatment specialization.

These indicators were evaluated separately by a diverse group of participants to determine their relevance and feasibility. The first group comprised seven members of involved project working groups who had participated in numerous bilateral meetings. The second group was made up of 18 members of the QM/RM board from German university hospitals and a third group consisted of a German-speaking panel of ten experts. This panel consisted of representatives from academia, patient advocacy groups, and health insurance companies, which will be described in step seven. During the development of the indicator set, the following criteria were applied to evaluate each indicator or recommendation (adapted from RAND*/ UCLA* Appropriateness Method [17]):

### Relevance

1. There is sufficient scientific evidence or professional consensus for the recommendation or indicator.
2. An improvement in patient care and staff safety is expected if the university hospital is organized or treats according to the recommendation or indicator.
3. University hospitals that are organized or treat according to the recommendation or indicator are considered high quality.

### Feasibility

1. The implementation of the recommendation or indicator is at the discretion of the university hospital.
2. The information or data necessary to verify the fulfillment of the recommendation or indicator is available in the university hospital.
3. The data documented in the university hospital concerning the recommendation or indicators are most likely accurate.
4. The absence of the corresponding information or data is fundamentally a sign of inadequate quality.

### Literature review (Fig. 1, step 1)

The literature search entailed an examination of relevant indicators on the websites of IGOs (World Health Organization (WHO), the Centers for Disease Control and Prevention (CDC), and the National Health Service (NHS). Additionally, national, and international indicator databases (Agency for Healthcare Research and Quality (AHRQ) and Google Scholar) were reviewed to identify relevant QIs. A targeted literature search was conducted on November 22, 2022, in PubMed. The search string can be found in Appendix A. Screening of both title and abstracts and full texts as well as data extraction were done by two persons from the Dresden and Marburg project site (JE and FW). No exclusion of records happened on the full-text level, however, indicators which were similar in contents were disregarded in the following analysis. Data extraction covered the indicators themselves as well as bibliographic information on their origin and the domains they covered, such as nosocomial infections and continuity of care.

The results were further consolidated in three interdisciplinary, bilateral meetings between the Dresden and Marburg project sites by using the criteria of relevance and feasibility mentioned above [18]. Where members of the project sites disagreed during the consolidation process, discrepancies were resolved through discussion or by involving one of the project’s principal investigators.

### Interviews (Fig. 1, step 1)

In order to incorporate firsthand experience from QM/RM officials during the pandemic into the core set, also in step one, three semi-structured qualitative interviews were conducted [19, 20]. The interviewees were selected from QM/RM departments of the sites involved in our project, ultimately leading to interviews with staff members from three university hospitals. These interviews took place in December 2022 using an online video conferencing tool and aimed to capture the unique experiences of these departments during the COVID-19 pandemic. The interviews were recorded if so consented to by the participants before the start of each interview, with each session lasting approximately one hour. They were conducted by a staff member from the Marburg site and documented by two additional staff members.

The interviews focused on exploring the impact of the pandemic on the quality of care and risks for patient safety at the university hospitals in Germany. Specific questions concerned observed declines in quality and increases in risks to safety, methods employed for their measurement or detection, actions taken to maintain standard care, case transfers to other hospitals, and lessons learned for the future. The recorded interviews were transcribed and transcripts served as the basis for analysis by three researchers (JE, NA, CM), with each interview being individually analyzed for recurring issues that constituted themes and could be used to extract relevant indicators (Fig. 1, step 2) [21]. A copy of the interview guideline is included in Appendix B.

### Evaluation of the Indicator Set (Fig. 1, step 3)

Based on the results of the literature and database search, as well as the interviews, an initial proposal for a core QI-set was created in several pretest phases: Initially, it was tested for functionality and comprehensibility internally at the Marburg Institute, followed by further tests with partners in the study group (Fig. 1, step 4). Testing for functionality and comprehensibility solely addresses the way each indicator is formulated, such making sure its scope is easily intelligible (comprehensibility) and it fits into the flow of the indicator checklists (functionality). Such tests of content validity are the gold standard in questionnaire development [22] and were considered essential prerequisite before the indicators were compiled into a survey (see next chapter). After each testing phase, adjustments were made to optimize the indicator set’s functionality and comprehensibility, drawing upon the expertise garnered from the predecessor project, egePan (Evidenzgeleitetes Pandemiemanagement) [8], and preliminary discussions with QM/RM officials (as described above). To further consolidate and clarify the indicators, several sessions were held within the project team in which these indicators were compared and integrated, with a specific focus on evaluating them according to their relevance and feasibility as mentioned above. The optimized indicator set was then transferred into an online survey.

### Online Survey of QM/RM Departments at German University Hospitals (Fig. 1, step 4)

A survey was designed to assess once more the relevance and feasibility of the indicators developed. The final version of the questionnaire was provided as an online survey (SoSci Survey platform [23]) and sent to all QM/RM departments of the German university hospitals on March 6, 2023, irrespective of their involvement in the indicator development process. Reminders were sent on March 21 and April 12. The estimated time to complete the questionnaire was 40–60 min. Questions were included to additionally inquire whether the individual indicators should be collected during and/or between pandemics.

The relevance and feasibility of the indicators were to be rated by the participants on a scale from one (not at all relevant/not feasible) to four (very relevant/easily feasible). Additionally, there was an option to comment on each indicator. The results were reviewed, analyzed, and evaluated by the project group (Fig. 1, step 5), leading to minor adjustments (Fig. 1, step 6, milestone). A copy of the questionnaire is included in Appendix C.

### Online Conference for the Evaluation and Finalization of the Proposed Core QI-set (Fig. 1, step 7)

On December 14, 2023, an online conference was held with members of an expert panel and all involved project partners of our study group, aiming to finally evaluate the core QI-set. The panel included members from various German institutions, e.g. the Institute for Quality Assurance and Transparency in Healthcare (IQTIG), the Department of Patient Safety at the University Hospital Bonn, the Institute for Quality & Patient Safety (BQS), the Competence Center for Quality Assurance (KCQ), the Patient Representation of the Federal Joint Committee (G-BA), the Working Group on Quality and Patient Safety Research (QPSF) of the German Network for Health Services Research (DNVF), the Academy for Public Health (AÖGW), and Germany’s second largest statutory health insurance agency (Techniker Krankenkasse). During the conference, the most up to date variant of the indicator set was once again discussed indicator by indicator. This led to minor changes such as adjustment of the numerators and denominators to legal regulations or standardization of terms. The finalization of the core QI-set was completed by incorporating agreed-upon changes following the online conference (Fig. 1, step 8). The final dataset (Fig. 1, step 9, third milestone) is included in Appendix D.

## Results

### Strand A—Organizational structure/management indicators

For Strand A, a total of 364 indicators were evaluated, including 28 indicators from the predecessor project egePan [24], 167 from the literature search in PubMed, 90 from interviews with QM/RM officials of university hospitals, and an additional 79 indicators from international organizations such as the WHO, the CDC, and the NHS.

**Fig. 2:**
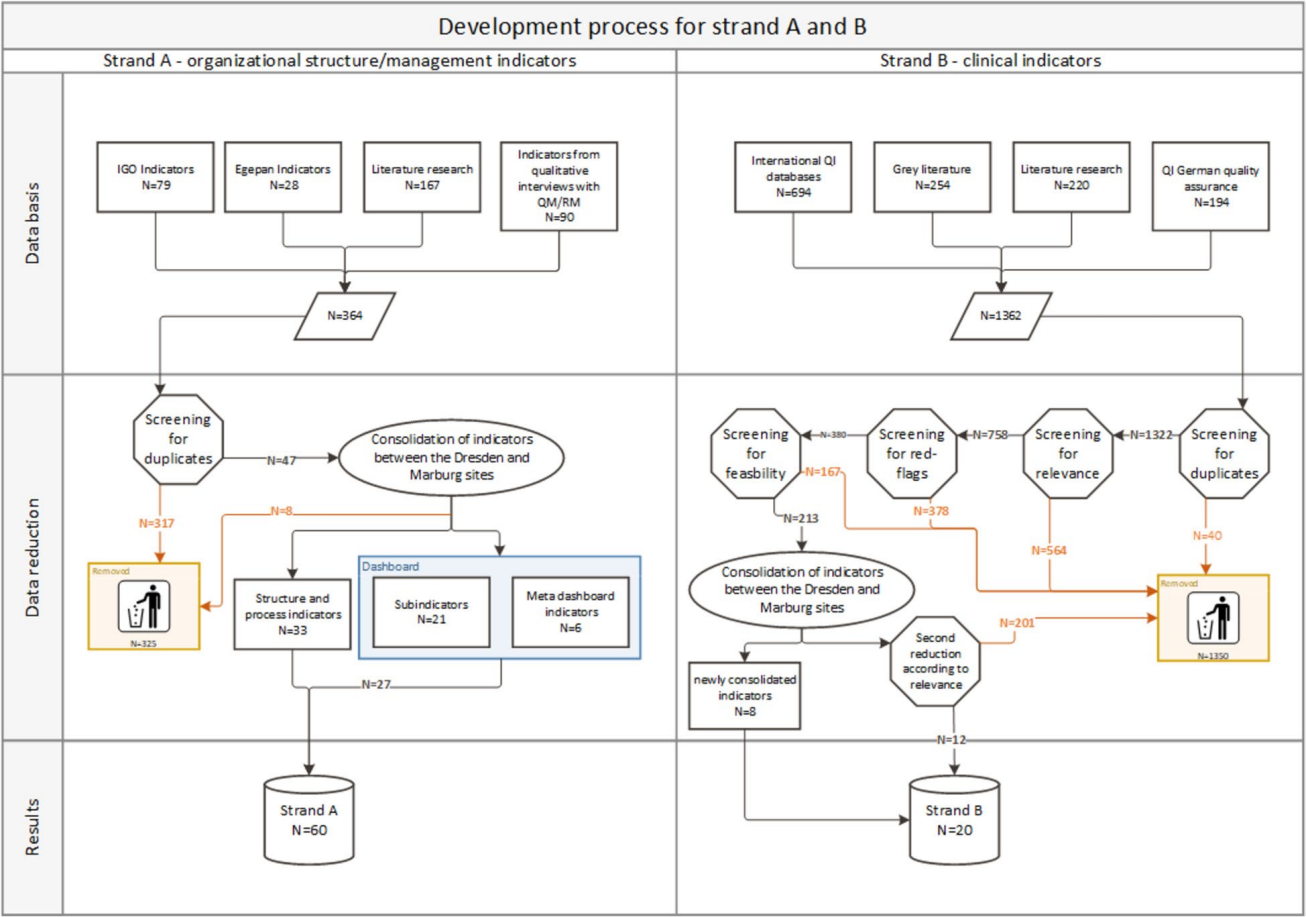
Development process for strand **A** and **B**. IGO = International/Governmental-Organizations, here WHO, CDC & NHS; egePan = Development, testing and implementation of regionally adaptive care structures and processes for evidence-based pandemic management coordinated by University Medicine; QM = Quality management; RM = Risk management; QI = Quality indicators; N = Numbers/Quantity

Subsequently, three research associates (JE, NA, CM) independently reviewed these 364 indicators, consolidated those that were identical, and reduced the list to 47 indicators. Following further expert evaluation of the QIs relevance and feasibility, eight indicators were rejected, leaving a dataset of 33 indicators. This dataset was supplemented by indicators related to bed and personnel management, which, according to the QM/RM officials consulted again on this list, proved to be essential for daily management during the pandemic. This resulted in 6 overarching indicators, to which further 21 detailed sub-indicators were defined as individual measurement parameters. These 27 indicators should be collected daily and are best presented in the form of a dashboard. This dashboard is intended to help continuously monitor metrics for potential threats and react quickly if necessary, such as in the case of staffing shortages. Figure 2 depicts the precise development process, including all steps of QI reduction.

### Strand B—Specific indicators for monitoring clinical quality and patient safety

The focus of Strand B was on disease-specific indicators related to selected exemplary diagnoses (so-called tracers), which include significant conditions such as stroke, myocardial infarction, and cancer. The tracer concept, developed by Kessner et al. [25], provides insights into the overall quality of medical care by focusing on exemplary diagnoses. Additionally, the medical areas of obstetrics and emergency care were identified as focal points since quality monitoring during a pandemic is of outstanding importance in these areas as well.

The literature search described above lead to the identification of a total of 1.362 QIs for Strand B. In the next step, 40 duplicates were identified and excluded, reducing the QI number to 1.322. Of these, 758 QIs were considered particularly relevant for patient safety and thus essential for ensuring regular medical care, following discussions within the Marburg team. Among these QIs, 380 were classified as critical "Red Flag Events", where a high degree of non-compliance can lead to severe medical errors as well as patient safety incidents [26]. In a further step of reduction, the study group assessed the feasibility of the indicators, resulting in 213 indicators being classified as 'feasible' at the end of this process. The list of the 213 QIs was once again consolidated with the working group from Dresden, reducing it to a final selection of 20 QIs. Of these, 12 were retained without modification, while eight were newly consolidated. The latter group comprised QIs incorporating content from international literature that needed to be adapted to German quality measurement standards. The guiding principles for this consolidation process were the fit with the tracer concept and the search for indicators already established in the German healthcare system for which no separate data collection would be necessary. Figure 2 depicts the exact development process.

### Evaluation

The questionnaire of the online survey, devised from the meticulously assembled set of indicators outlined above (first milestone), was divided into the areas of organizational structure/management, and quality and patient safety. The questionnaire was sent to the QM/RM departments of all 36 university hospitals, where it was started 48 times in total, with 18 individuals completing it fully and 29 completing it partially. Participants had the option to indicate their location, a choice not utilized by the majority, resulting in the survey remaining largely anonymous. All responses, including those from partially completed questionnaires, were included in the analysis to capture a comprehensive view of opinions and ratings.

All indicators in the organizational structure/management area were rated positively in terms of their relevance (see the full list of indicators in Appendix D). Three specific indicators from this segment were deemed not feasible:

- QI 10: Use of a digital dashboard with all essential control information, like the number of admissions & discharges in general wards, separated by pandemic and other patients or the total proportion of available nursing staff per patient, and functions (62.5% negative votes concerning feasibility, *n* = 16).
- QI 17: Planning alternative procurement sources for materials relevant during pandemic times (55.6% negative votes, *n* = 18).
- QI 19: Regular training of non-patient-facing staff with potentially relevant qualifications for dealing with patients and processes in pandemic times (77.8% negative votes, *n* = 18).

Furthermore, two indicators from the quality and patient safety indicators area were predominantly assessed as of little relevance in pandemic situations:

- QI 48: Recording the number of inpatient-acquired fall-induced fractures (58.8% negative votes concerning relevance, *n* = 17).
- QI 62: Recording the number of inpatient admissions with various mental illnesses per week (68.8% negative votes, *n* = 16).

Another indicator from the quality and patient safety area was predominantly deemed not feasible:

- QI 57: Recording the number of cancer patients in whom diagnosis or therapy was delayed due to hospital reasons (56.3% negative votes concerning feasibility, *N* = 16)

Based on this evaluation, the core QI-set was re-evaluated within the study group. It was noted that comments from QM/RM officials like “Are indeed recorded via hospital hygiene.” or “What is the relation to the pandemic, quality assurance data?” on indicators negatively assessed in terms of relevance and feasibility suggested a misunderstanding of the basic concept. Within the questionnaire, respondents repeatedly commented that indicators meant to assess the pandemic situation are already being captured, leading to these indicators being mistakenly seen as "duplicated" by the respondents and therefore rejected.

Based on the insights from the survey and subsequent evaluation, the indicators were supplemented with a rough definition of the respective numerators and denominators (second milestone), linguistically rephrased, and the core dataset was restructured and divided into the following categories, all by the research team:

- Organizational Structure / Management Indicators
- Personnel Management Indicators
- Clinical Quality and Patient Safety Indicators
- Indicators for a Pandemic Dashboard

Afterwards, the current form of the indicators, after evaluation by the QM/RM members of the university hospitals, was discussed individually in an online meeting of the mentioned expert group, leading to minor adjustments (third milestone). In the category *Personnel Management Indicators*, the panel added the introduction of full-time equivalents for both numerator and denominator; for Indicator 46. *Number of postponed procedures*, a more precise denominator and the concept of service groups were added to the framework. Furthermore, guided by the panel's suggestions, three novel indicators were introduced, focusing on the composition of teams, cesarean delivery rates, and the criteria for admitting new patients.

### Monitoring

In the subsequent sections, specific examples of these indicators and recommendations for their measurement will be presented. The overarching goal of the monitoring effort is to preserve the quality and safety of medical care during pandemic circumstances and to enhance preparedness for future pandemics. Ultimately, this initiative seeks to facilitate long-term inter-university hospital benchmarking. Examples for all the following indicator categories with numerator and denominator can be taken from Table 1, a complete indicator list is located in Appendix D.

**Table 1:** Examples of indicators from the respective categories

	**Numerator**	**Denominator**
**Organizational Structure / Management Indicators (collected via self-report / peer review / audit; semi-/annually)**		
Pandemic team / crisis management established (including pandemic coordinator, hospital management, QM/RM, hygiene officer, ICU director, emergency director, infectious disease specialist/epidemiologist, technical services officer, procurement, disaster management (external contacts), pharmacy, occupational health, press spokesperson, and project manager)	yes/no	Hospital
Tasks for each team member defined (including deputy, to ensure implementation even in case of absence [professional representation] and to maintain interdisciplinarity)	yes/no	Hospital
Maintain and update contact lists of employees including qualification (for all hierarchical levels)	yes/no	Hospital
[…]	[…]	[…]
**Personnel Management Indicators (daily collection)**		
Staff availability by qualification and ward/department	Actual available staff by qualification and ward/department	Potential staff (Number + FTE) by qualification and ward/department
Number of employees by ward and qualification	Number of doctors, nursing staff, nursing assistants (+ FTEs)	Per ward
Proportion of employees on vacation by ward and qualification	Number of employees on vacation (+ FTEs) by ward and qualification	Number of all employees per ward and qualification
[…]	[…]	[…]
**Clinical Quality and Patient Safety Indicators (collected weekly, quarterly)**		
Overall hospital mortality/by department	Deaths per week / in the department	All cases / per department per week
Number of postponed indicated procedures / by service group	Diagnostic or therapeutic procedures delayed by at least one day	Scheduled diagnostic or therapeutic procedures per day and department and service group
Number of unplanned readmissions (Ratio)	Number of unplanned readmissions per week	Number of discharges per week
[…]	[…]	[…]
**Indicators for a Pandemic Dashboard; continuous monitoring during pandemics, regular practice of "in-line" data collection**		
Regional pandemic incidence	New cases per day	Regional population (per 100,000)
Hospitalization rate / hospital incidence	Hospital admissions of infected individuals per day	Infected per 100,000
Number of occupied beds by category, with staffing threshold in the hospital and department, separated by pandemic and other patients	Beds occupied with pandemic/non-pandemic patients per department	All beds for which staff is available by department
[…]	[…]	[…]

*Organizational Structure / Management indicators*, include recommendations appropriate for pandemics, which could be reviewed semi-annually through self-assessment and reporting, and annually through peer review or audit with other service providers in an open benchmarking process. *Personnel Management Indicators* should typically be collected daily from personnel management, which are often only available in a de-centralized manner but must be made available centrally for management in pandemic situations. *Clinical Quality and Patient Safety Indicators* are particularly relevant under pandemic conditions for patients not affected by the pandemic (patient endangerment due to organizational overload, time-critical treatments, hygiene). Under pandemic conditions, a weekly "in-line," monthly, or quarterly evaluation, depending on the frequency of measurement for the content would be recommended. The fourth category includes *Indicators for a Pandemic Dashboard*, that must be monitored promptly/daily or continuously under pandemic conditions. Data collection for all these indicators should be regularly practiced enabling daily collection during pandemics. Where available, indicators already captured by the statutory quality assurance system in Germany were used. Examples of the 83 indicators can be viewed in tables 1–4, the full set can be found in the Appendix D.

## Discussion

The development and refinement of QIs for managing organizational structure and clinical quality and patient safety under pandemic conditions, as detailed in our results, mark a crucial step forward in the operational preparedness of university hospitals for crisis situations. The core QI-set represents a further development of the pandemic-related indicator collection already published as part of the University Medicine Network (NUM) [9, 24]. It fits with the general objective of the NUM to strengthen the pandemic preparedness of German university medicine. The extensive evaluation process, culminating in a comprehensive set of indicators, reflects the guidance provided by international health organizations [10–13] for health emergencies like a pandemic.

The reduction of initial indicators to a core set as shown above, including both general organizational and disease-specific indicators, recognizes the importance of methodologically sound approaches to assess healthcare quality. The use of the tracer concept for selecting diseases [25] allows for an in-depth evaluation of healthcare quality across crucial medical conditions, potentially offering insights into the broader implications for patient safety and healthcare delivery during pandemics.

The establishment of a dashboard for continuous monitoring of key indicators is an innovative solution to the dynamic challenges posed by pandemics. This tool facilitates real-time decision-making and rapid response to emergent issues, such as staffing shortages, critical for maintaining service continuity and quality during crises, reinforcing the need for preparedness as outlined by sources like the CDC [10]. Broader frameworks of pandemic preparedness have since identified monitoring and surveillance as an essential prerequisite of ensuring quality of health care during a pandemic [27]. Successful initiatives in regional care coordination have mostly relied on dashboards assuring real-time data provision as well [14, 15].

Feedback from the online survey and expert panel meetings highlighted the need for clarity in the presentation and understanding of pandemic-specific QIs. This underscores the importance of effective communication and training within healthcare institutions, echoing the sentiments of healthcare professionals during the early stage of the COVID-19 pandemic in Germany [5] and the strategic planning efforts coordinated by institutions of university medicine [8]. Addressing the identified gaps in feasibility and relevance through reevaluation and refinement of the indicators is a responsive and adaptive approach to quality management in unprecedented situations.

The study's focus on the German university hospital system offers invaluable insights. The applicability of the findings to other healthcare institutions, however, may be contingent upon local healthcare structures and pandemic response strategies.

In one of the steps taken to develop the core QI-set, only *n* = 18 questionnaires were completed, indicating a potential limitation in the breadth of data collected. The feasibility of using the indicator set in everyday health care routines remains to be assessed in a subsequent phase, underscoring the need for future research to evaluate the implementation and impact of the proposed quality indicators and dashboard in real-world settings. Such research should particularly focus on their effectiveness in enhancing patient care, staff safety, and overall hospital resilience. The development of our indicator set involved compiling individual indicators from various sources, as no comprehensive set of this kind existed prior to our work. While international literature was used to identify relevant indicators, a complete set like ours had not been reported elsewhere. This underscores the unique composition of our work and highlights its contribution to healthcare quality improvement, especially in offering a basis for future international benchmarks.

## Conclusion

Our study contributes to the growing body of knowledge on healthcare quality management during pandemics, offering a comprehensive set of QIs that reflect both organizational and clinical priorities. By facilitating a structured approach to pandemic preparedness and response, these indicators have the potential to significantly enhance the resilience and responsiveness of university hospitals and other healthcare institutions facing similar challenges.

## Supplementary Information

Supplementary Material 1.

Supplementary Material 2.

Supplementary Material 3.

Supplementary Material 4.

## Data Availability

The search string for the literature review, the interview guide, the questionnaire for assessing relevance and feasibility for the quality indicators, as well as the final list of indicators are available in the digital appendix.
