# Supplementary material for: Development of a pandemic-related core set of quality indicators for quality and patient safety in University Hospitals in Germany
Source: BMC Health Serv Res. 2025 Jan 8;25:43. doi: 10.1186/s12913-024-12194-3 (PMC11708090; doi:10.1186/s12913-024-12194-3)
Supplement: Supplementary file 1 — Supplementary Material 1. [file 12913_2024_12194_MOESM1_ESM.docx]

**Appendix A – Search String**

(("covid-19"[Title/Abstract]) AND ("hospital"[Title/Abstract])) AND ((("risk management"[Title/Abstract]) OR ("patient safety"[Title/Abstract])) OR ("quality of health care"[Title/Abstract])).
