# Supplementary material for: Development of a pandemic-related core set of quality indicators for quality and patient safety in University Hospitals in Germany
Source: BMC Health Serv Res. 2025 Jan 8;25:43. doi: 10.1186/s12913-024-12194-3 (PMC11708090; doi:10.1186/s12913-024-12194-3)
Supplement: Supplementary file 2 — Supplementary Material 2. [file 12913_2024_12194_MOESM2_ESM.docx]

**Appendix B – Interview Guide**
**Guide: Expert Interview AP4**
**Structure of the Guide:**

- **Narrative prompts (1–3)**
- **Evaluation questions (4–6)**
- **Attitudinal questions (7–8)**

| **Topics** | **Keywords and Possible Follow-Up Questions** | **Notes** | **Possible Indicator(s)** |
| --- | --- | --- | --- |
| **Brief Introduction of the Participants** | "Please introduce yourself briefly. What is your position, and what are your responsibilities?" |  |  |
| **1. Explanation of the Research Topic** | During the COVID-19 pandemic, university hospitals experienced a decline in the utilization of inpatient care by patients with urgent medical needs unrelated to COVID. Acutely ill patients were often admitted only at a critical stage of their illness. Additionally, many university hospitals had fully utilized their intensive care capacities, leading to competition for these resources and earlier discharges to general wards or outpatient care. These developments resulted in diminished quality of care and increased risks for patients. |  |  |
| **2. What experiences did you have regarding reduced care quality and the associated increased safety risks in your hospital?** | "We would like to hear how your institution managed non-COVID patients."  "Were processes disrupted and/or delayed?"  "What were the most significant restrictions in treatment?" |  |  |
| **3. In which specific areas did quality reductions and risks for patients occur?** | "Which patient groups and/or diagnostic or therapeutic procedures?"  "What about hospital staff?" |  |  |
| **4. How were these issues measured or assessed? Alternatively: How were the safety and quality limitations noticed?** | "Via QI (Quality Indicators), frequencies, metrics, etc.?"  "If not, why? What challenges/obstacles existed? Would you have liked to measure them?" |  |  |
| **5. What measures were taken or derived from the data to maintain regular care?** | "How was the implementation carried out in practice?"  "Would you describe the measures taken as effective?" |  |  |
| **6. Were cases transferred to or outsourced to other hospitals?** | "Which cases?"  "Was there a prioritization list or similar? (What was the reasoning?)"  "Was follow-up care still provided or supervised by your hospital?" |  |  |
| **7. Now, after experiencing multiple COVID waves, what would you do differently to ensure care quality and minimize risks?** | "Do you have suggestions for improvement?" |  |  |
| **8. Conclusion** | "Would you like to add anything on this topic?"  "Could you provide us with the prioritization list, QI data, or other mentioned documents?" |  |  |
