# Supplementary material for: Development of a pandemic-related core set of quality indicators for quality and patient safety in University Hospitals in Germany
Source: BMC Health Serv Res. 2025 Jan 8;25:43. doi: 10.1186/s12913-024-12194-3 (PMC11708090; doi:10.1186/s12913-024-12194-3)
Supplement: Supplementary file 3 — Supplementary Material 3. [file 12913_2024_12194_MOESM3_ESM.docx]

**Quality Management and Clinical Risk Management in the Context of Pandemic Management (PREPARED)**

During the COVID-19 pandemic, there was a decline in the utilization of university hospitals, which affected not only planned elective hospital stays but also inpatients with an urgent need for care unrelated to COVID. Acutely ill patients were often admitted only at a critical stage of illness. Additionally, the intensive care capacities of many university hospitals were fully utilized, leading to competition for intensive care resources and earlier discharges to normal wards or aftercare. These developments result in a reduction in quality and increased risks for patients. To identify such risks early, continuous monitoring of quality and risks is necessary.

In this context, the project "Quality Management and Clinical Risk Management" as part of the PREPARED project of the Network University Medicine (NUM), led by the cooperation partners Institute for Healthcare Research and Clinical Epidemiology of the University of Marburg, Center for Evidence-Based Healthcare (ZEGV), University Hospital and Medical Faculty Carl Gustav Carus of the Technical University of Dresden, as well as the Central Department of Quality Management and Clinical Risk Management of the University Hospital Essen, has the task of developing a concept for quality and risk management. This concept is to be used by all university hospitals in Germany in the future to maintain the quality and safety of patient care and employee safety under pandemic conditions.

Based on a literature review and a survey of the participating university hospitals, a list of recommendations and indicators has been created for which we would like to gather your opinion. We kindly ask you to assess the relevance and feasibility of the recommendations or indicators for quality and risk management in the event of a pandemic (or in preparation for a pandemic). Completing the anonymous survey takes approximately 45 minutes and can be interrupted and continued later if desired. The name of the university hospital is provided for completeness only. In the presentation of results, only pseudonymized data will be used, so no individual university hospitals will be named.

**Instructions for Completing the Questionnaire**

The following questionnaire contains 56 recommendations and indicators that we ask you to evaluate in terms of relevance and feasibility. The evaluation is done on a scale of 1 to 4, with 4 indicating the highest relevance and easiest feasibility, and 1 indicating no relevance or not feasible. You must answer both scales for each indicator and recommendation. Please also indicate whether the indicator should be measured during a pandemic and/or between pandemics.

The resulting recommendations and indicators can be divided into three sections:

- Indicators and recommendations for preparing for and maintaining hospital operations in a pandemic situation
- Indicators and parameters of a dashboard for managing processes during a pandemic situation

Indicators for the continuous examination of treatment quality and safety risks

To increase the comparability of the assessment, we would like you to evaluate the relevance and feasibility of the recommendation based on the following definitions of the two criteria:

**Relevance**

1. In your opinion, there is sufficient scientific evidence or professional consensus for the recommendation or indicator.
2. If the university hospital is organized or treats accordingly, better and safer patient care and employee safety can be expected.
3. University hospitals that are organized or treat accordingly are generally considered to be of high quality.

**Feasibility**

1. The implementation of the recommendation or indicator is in the hands of the university hospital.
2. The information or data necessary to verify the fulfillment of the recommendation or indicator is available in the university hospital.
3. The data documented in the university hospital about the recommendation or indicators is most likely correct.
4. The lack of corresponding information or data is basically a sign of poor quality.

|  | Quality Indicator | Additional Information | Relevance | Feasibility | When should the QI be measured? |
| --- | --- | --- | --- | --- | --- |
| Structural Indicators | | | | | |
| 1. | Pandemic team / crisis management team established | e.g., pandemic officer, hospital management, QM/RM, hygiene officer, ICU head, emergency head, infectious disease specialist/epidemiologist, technical services officer, procurement, disaster protection (external contacts), pharmacy, occupational medicine, press officer, and project manager | 1-4 | 1-4 | During the pandemic / Interpandemic phase / Both |
| 2. | Tasks of each team member defined | Including deputy to ensure implementation even in case of absence [professional representation] and to maintain interdisciplinarity | 1-4 | 1-4 | During the pandemic / Interpandemic phase / Both |
| 3. | Maintain and update contact lists of employees including qualification information | for all hierarchy levels | 1-4 | 1-4 | During the pandemic / Interpandemic phase / Both |
| 4. | Establish and maintain regular meeting rhythm | Meetings of the pandemic commission, in specialist departments and wards, also continue under regular rhythm for patient-relevant discussions in care under pandemic conditions (e.g., through alternative communication methods [such as video conferences, etc.]) | 1-4 | 1-4 | During the pandemic / Interpandemic phase / Both |
| 5. | Information media and communication channels for current and continuous information dissemination under pandemic conditions | From the management level to all other levels | 1-4 | 1-4 | During the pandemic / Interpandemic phase / Both |
| 6. | Establish information media and communication channels for current and continuous feedback under pandemic conditions | From the lower levels to the management level | 1-4 | 1-4 | During the pandemic / Interpandemic phase / Both |
| 7. | Maintain a comprehensive pandemic chronicle | This includes, among other things, all relevant decisions with justifications (protocols, repurposing, ward closures, process changes, photo and error database, ...) | 1-4 | 1-4 | During the pandemic / Interpandemic phase / Both |
| 8. | Obtain and coordinate information on the status quo and prognosis of expected disease cases, protective equipment, bed, and treatment capacity with surrounding providers |  | 1-4 | 1-4 | During the pandemic / Interpandemic phase / Both |
| 9. | Maintain structures and processes for the transfer of resources, staff, and patients between facilities | Define thresholds for the number and severity of patients beyond which care is no longer possible in one's own facility – coordination with surrounding providers | 1-4 | 1-4 | During the pandemic / Interpandemic phase / Both |
| 10. | Establish alternative communication channels to ensure contact with and between patients, doctors, and relatives during the treatment process under pandemic conditions | Alternative to personal meetings, such as via video telephony | 1-4 | 1-4 | During the pandemic / Interpandemic phase / Both |
| 11. | Pandemic-adapted complaint management system | Ensure patient access to complaint management | 1-4 | 1-4 | During the pandemic / Interpandemic phase / Both |
| 12. | Regular evaluation of the CIRS system | Monitor and analyze reported events per week in the CIRS system | 1-4 | 1-4 | During the pandemic / Interpandemic phase / Both |
| 13. | Define separate areas for the access and care of pandemic and non-pandemic patients | Define wards that can be repurposed for a pandemic at short notice to ensure separation of pandemic and non-pandemic patients, preferably without the complete closure of repurposed areas/wards | 1-4 | 1-4 | During the pandemic / Interpandemic phase / Both |
| 14. | Prioritize non-pandemic-related treatments that need to be maintained | Elective/subacute*/emergency (*=not life-threatening, but for prognostic reasons (pain, mobility, quality of life, survival probability, chronicity, etc.), timely treatment is important) | 1-4 | 1-4 | During the pandemic / Interpandemic phase / Both |
| 15. | Determine the resources necessary for the care of pandemic patients | Plan personnel and materials adapted to the pandemic situation | 1-4 | 1-4 | During the pandemic / Interpandemic phase / Both |
| 16. | Determine the resources necessary for the care of non-pandemic patients | Plan personnel and materials adapted to the pandemic situation | 1-4 | 1-4 | During the pandemic / Interpandemic phase / Both |
| 17. | Determine the resources necessary for the care of pandemic patients | Particularly for intensive care capacities | 1-4 | 1-4 | During the pandemic / Interpandemic phase / Both |
| 18. | Plan alternative procurement options/sources for pandemic-related materials | Consider various suppliers | 1-4 | 1-4 | During the pandemic / Interpandemic phase / Both |
| 19. | Check the quality of supplied materials (such as protective equipment) |  | 1-4 | 1-4 | During the pandemic / Interpandemic phase / Both |
| 20. | Maintain a training concept for repurposed personnel and train and test employees as needed | Retrained personnel should be demonstrably familiar with the requirements of the new work environment, for example, when moving from normal to intensive care units | 1-4 | 1-4 | During the pandemic / Interpandemic phase / Both |
| 21. | Regularly train personnel not directly involved with patients but who have qualifications useful during the pandemic in handling patients and processes | Regularly train personnel with nursing qualifications but no longer working in active nursing to keep them ready for crisis situations | 1-4 | 1-4 | During the pandemic / Interpandemic phase / Both |
| 22. | Establish a crisis intervention team for employees | A low-threshold offer for psychosocial support for employees exists | 1-4 | 1-4 | During the pandemic / Interpandemic phase / Both |
| 23. | Provide pandemic-compliant accommodation and catering options for employees | employees Provision of food (compensating for closed tea kitchens, canteen, cafeteria), accommodation options | 1-4 | 1-4 | During the pandemic / Interpandemic phase / Both |
| 24. | Define standards for early discharge/transfer | Ensure that patients are not discharged in a critical/uncertain condition and/or into a potentially dangerous situation | 1-4 | 1-4 | During the pandemic / Interpandemic phase / Both |
| 25. | Record and document patients' will | For potentially necessary intensive treatment | 1-4 | 1-4 | During the pandemic / Interpandemic phase / Both |
| 26. | Plan handling of deceased patients | Especially in situations of increased burden | 1-4 | 1-4 | During the pandemic / Interpandemic phase / Both |
| 27. | Record infectivity in employees (infection outside/infection in the hospital), patients, and visitors | Conduct regular, pandemic-adjusted tests in patient/employee contact | 1-4 | 1-4 | During the pandemic / Interpandemic phase / Both |
| 28. | Introduce infection prophylaxis for employees | For example, through informational events, free rapid tests, masks, and vaccination | 1-4 | 1-4 | During the pandemic / Interpandemic phase / Both |
| 29. | Establish standardized procedures for pandemic and non-pandemic patients in shared areas | For example, distance, medical mask, sequence in appointment scheduling, disinfection | 1-4 | 1-4 | During the pandemic / Interpandemic phase / Both |
| 30. | Inform visitors and patients about special hygiene measures | Ensure that the information is always up to date | 1-4 | 1-4 | During the pandemic / Interpandemic phase / Both |
| 31. | Permanently and regularly control preventive hygiene measures and behavioral rules | This includes, in particular, the continued use of protective clothing, masks, tests, distancing within the building, regular ventilation, and hand hygiene – regardless of the severity of the pandemic situation outside the facility and the duration of the pandemic | 1-4 | 1-4 | During the pandemic / Interpandemic phase / Both |
| 32. | Establish visitation rules and contact tracing for visitors and suppliers | To clarify infection chains | 1-4 | 1-4 | During the pandemic / Interpandemic phase / Both |
| 33. | Use a digital dashboard with all necessary information and functions for management |  | 1-4 | 1-4 | During the pandemic / Interpandemic phase / Both |
| Clinical Indicators | | | | | |
| 1. | Record hospital mortality by department | across facilities | 1-4 | 1-4 | Only during the pandemic |
| 2. | Record the number of postponed indicated procedures | across facilities | 1-4 | 1-4 | Only during the pandemic |
| 3. | Record the number of unplanned readmissions | across facilities | 1-4 | 1-4 | Only during the pandemic |
| 4. | Record the number of catheter-associated infections (vessels, urinary tract) | across facilities | 1-4 | 1-4 | Only during the pandemic |
| 5. | Record the number of hospital-acquired fracture-related falls | across facilities | 1-4 | 1-4 | Only during the pandemic |
| 6. | Record the number of hospital-acquired pulmonary embolisms across facilities | across facilities | 1-4 | 1-4 | Only during the pandemic |
| 7. | Record hospital-acquired decubitus ulcers (excluding stage/category 1) | Skin damage | 1-4 | 1-4 | Only during the pandemic |
| 8. | Record hospital mortality in full-term live births | Obstetrics | 1-4 | 1-4 | Only during the pandemic |
| 9. | Record decision-to-delivery time (D-D time) over 20 minutes in emergency cesarean sections | Obstetrics | 1-4 | 1-4 | Only during the pandemic |
| 10. | Monitor tumor stage at first presentation of patients | Oncology | 1-4 | 1-4 | Only during the pandemic |
| 11. | Record the number of patients admitted with a cancer diagnosis per week | Oncology | 1-4 | 1-4 | Only during the pandemic |
| 12. | Anzahl an Krebspatient:innen, deren Diagnostik oder Therapie kliniksbedingt verzögert stattfindet, erfassen | Onkologie | 1-4 | 1-4 | Only during the pandemic |
| 13. | Record "door-to-balloon" time for first PCI with ST-elevation myocardial infarction indication | Cardiology \| <= 60 minutes after admission | 1-4 | 1-4 | Only during the pandemic |
| 14. | Record the time in the emergency department until the decision on further treatment location/transfer | Emergency department | 1-4 | 1-4 | Only during the pandemic |
| 15. | Record the number of overload reports per day | Personnel | 1-4 | 1-4 | Only during the pandemic |
| 16. | Record the number of suicides and suicide attempts during hospitalization | Psychiatry | 1-4 | 1-4 | Only during the pandemic |
| 17. | Record the number of inpatient admissions with various mental illnesses per week | Psychiatry | 1-4 | 1-4 | Only during the pandemic |
| 18. | Record time to first imaging | Stroke care \| <= 30 minutes after admission | 1-4 | 1-4 | Only during the pandemic |
| 19. | Record nosocomial, postoperative wound infections after inpatient operations (non-implant operations) | Surgical | 1-4 | 1-4 | Only during the pandemic |
| 20. | Record nosocomial, postoperative wound infections after inpatient operations (implant operations) | Surgical | 1-4 | 1-4 | Only during the pandemic |

Dashboard Indicators

- **Do you already use a dashboard?**
- Yes
- No
- No, but we would like to work with a dashboard
- Not yet, but an introduction is planned

**Which of the following contents should be displayed on a dashboard?**

Please check all indicators that should be measured on a dashboard (Multiple answers possible):

- Regional pandemic incidence
- Pandemic hospitalization rate/hospital incidence
- Forecast of expected cases by ward (normal ward, intensive care unit, requiring ventilation, requiring ECMO)
- Forecast of expected occupancy rates on the respective wards
- Mortality rate by hospital and department
- Staff availability by qualification and ward/department
- Number of occupied beds by category, with staffing thresholds in the hospital and departments, separated by pandemic and non-pandemic patients

**Which parameters should be collected daily for the dashboard?**

Please check all parameters that should be collected daily:

- Number of pandemic and non-pandemic patients in the hospital
- Number of admissions & discharges by ward in the hospital, separated by pandemic and non-pandemic patients
- Number of pandemic and non-pandemic patients in the normal wards
- Number of admissions & discharges in the normal wards, separated by pandemic and non-pandemic patients
- Number of admissions & discharges in the intensive care units, separated by pandemic and non-pandemic patients
- Number of pandemic and non-pandemic patients in the intensive care units
- Number of staff by ward and qualification
- Proportion of pandemic and non-pandemic patients / normal beds
- Proportion of pandemic and non-pandemic patients / intensive care beds
- Proportion of intensive care patients infected with pandemic pathogens but not being treated in the intensive care unit because of the pathogen
- Proportion of repurposed pandemic beds occupied by pandemic patients
- Proportion of repurposed intensive pandemic beds occupied by pandemic patients
- Proportion of occupied isolation beds occupied by pandemic patients
- Proportion of total bed count occupied by pandemic patients
- Proportion of functional ventilators, separated by pandemic and regular operation
- Proportion of intensive care nurses / intensive care beds, separated by pandemic and non-pandemic patients
- Sick leave by qualification and ward
- Total proportion of sick staff
- Total proportion of available nursing staff per patient
- Proportion of staff on vacation by ward and qualification
- Total proportion of staff not present at work on the current day
