# Supplementary material for: Development of a pandemic-related core set of quality indicators for quality and patient safety in University Hospitals in Germany
Source: BMC Health Serv Res. 2025 Jan 8;25:43. doi: 10.1186/s12913-024-12194-3 (PMC11708090; doi:10.1186/s12913-024-12194-3)
Supplement: Supplementary file 4 — Supplementary Material 4. [file 12913_2024_12194_MOESM4_ESM.docx]

**Appendix D – Core set of QIs**

| **Organizational Structure / Management Indicators (collected via self-report / peer review / audit; semi-/annually)** | **numerator** | **denominator** |
| --- | --- | --- |
| 1. Establish a Pandemic / crisis management team (including pandemic coordinator, hospital management, QM/RM, hygiene officer, ICU director, emergency director, infectious disease specialist/epidemiologist, technical services officer, procurement, disaster management (external contacts), pharmacy, occupational health, press spokesperson, and project manager) | yes/no | Hospital |
| 1. Define tasks for each team member (including deputy, to ensure implementation even in case of absence [professional representation] and to maintain interdisciplinarity) | yes/no | Hospital |
| 1. Maintain and update contact lists of employees including qualification (for all hierarchical levels) | yes/no | Hospital |
| 1. Establish and maintain a regular meeting rhythm (meetings of the pandemic commission, in departments and wards, also allowing patient-relevant meetings in care under pandemic conditions to continue under regular rhythm [for example, through alternative communication methods like video conferences etc.]) | yes/no | Hospital |
| 1. Establish information media and channels for the current and continuous information dissemination under pandemic conditions (from the management level to all other levels and to municipal/state crisis management teams) | yes/no | Hospital |
| 1. Establish information media and channels for the current and continuous information feedback under pandemic conditions (from the lower levels to the management level and to municipal/state crisis management teams) | yes/no | Hospital |
| 1. Keep a complete pandemic chronicle (This includes all relevant decisions with reasons [minutes, repurposing, ward closure, process changes, photo and error database, ...]) | yes/no | Hospital |
| 1. Gather information on the current status and forecast of expected cases, protective equipment, bed and treatment capacity, and coordinate with surrounding service providers and municipal/state crisis management teams | yes/no | Hospital |
| 1. Establish alternative communication channels to ensure contacts with and between patients, doctors, and relatives during treatment under pandemic conditions (Alternative to personal conversation, for example via video telephony) | yes/no | Hospital |
| 1. Use a digital dashboard with all necessary control information and functions (Interactive interface that allows data to be visually presented and monitored to quickly capture and understand important information; provide access for municipal/state crisis management teams) | yes/no | Hospital |
| 1. Evaluate the CIRS system regularly (monitor and analyze events reported in the CIRS system per week) | yes/no | Hospital |
| 1. Establish a pandemic-adapted complaint management system (Ensure access to complaint management for patients) | yes/no | Hospital |
| 1. Define separate areas for access and care of pandemic and other patients (Define wards that can be repurposed for a pandemic at short notice and that ensure separation of pandemic and other patients, ideally without the closure of repurposed areas/wards) | yes/no | Hospital |
| 1. Determine the resources necessary for the care of non-pandemic patients (Planning of personnel and materials adapted to the pandemic situation) | yes/no | Hospital |
| 1. Determine the resources necessary for the care of pandemic patients (Planning of personnel and materials adapted to the pandemic situation, including personal protective equipment) | yes/no | Hospital |
| 1. Plan a graded repurposing of resources for pandemic needs (especially intensive care capacities) | yes/no | Hospital |
| 1. Plan alternative procurement options/sources for pandemic-relevant material (Provide for various suppliers) | yes/no | Hospital |
| 1. Check the quality of supplied materials (such as protective equipment) | yes/no | Hospital |
| 1. Have a training concept for repurposed personnel and train and test employees as needed (Repurposed personnel should demonstrably be familiar with the requirements of the new work environment, for example, when switching from regular to intensive care units) | yes/no | Hospital |
| 1. Regularly assess the team structure of newly formed teams (especially after transfers / reallocations) | yes/no | Hospital |
| 1. Establishment of a crisis intervention team for employees (There is a low-threshold offer for psychosocial support of employees) | yes/no | Hospital |
| 1. Define standards for early discharge/transfer (ensure that patients are not discharged in a critical/uncertain condition and/or into a potentially dangerous situation) | yes/no | Hospital |
| 1. Define standards for patient admission (ensure that urgently to be treated or patients in a critical condition are not turned away) | yes/no | Hospital |
| 1. Record and document patient will (for potentially necessary intensive care treatment) | yes/no | Hospital |
| 1. Prioritize non-pandemic-related treatment occasions whose care should be maintained (elective/subacute*/emergency: *=not life-threatening, but timely treatment is important for prognostic reasons (pain, mobility, quality of life, survival probability, chronicity, etc.)) | yes/no | Hospital |
| 1. Plan handling of deceased (especially in overload situations, Note: infection protection) | yes/no | Hospital |
| 1. Provide structures and processes for the transfer of resources, employees, patients between facilities (Define thresholds, for example, for the number and severity of patients, from which no care in one's own house is possible anymore – coordination with surrounding providers) | yes/no | Hospital |
| 1. Provide pandemic-compliant accommodation and catering options for employees (Catering with food (compensation for closed tea kitchens, canteen, cafeteria), accommodation options) | yes/no | Hospital |
| 1. Regularly train personnel deployed away from patients, with possibly useful qualifications in dealing with patients and processes during a pandemic (Staff who have nursing training but no longer work in active nursing service, regularly train to keep them ready for use in crisis situations) | yes/no | Hospital |
| 1. Establish visitation rules and contact tracing for visitors and suppliers (for clarifying infection chains) | yes/no | Hospital |
| 1. Inform visitors and patients about special hygiene measures (ensure that the information is always up-to-date and aligned with municipal requirements) | yes/no | Hospital |
| 1. Introduce infection prophylaxis for employees (for example, through information events, free rapid tests, masks, and vaccination) | yes/no | Hospital |
| 1. Capture infectivity among employees (infection outside/hospital), patients, and visitors (Conduct regular, pandemic-adapted tests in patient/employee contact) | yes/no | Hospital |
| 1. Establish standardized procedures for pandemic and other patients in commonly used areas (for example, distance, medical mask, order in appointment scheduling, disinfection) | yes/no | Hospital |
| 1. Permanently and regularly control preventive hygiene measures and rules of conduct (includes especially the continued use of protective clothing, masks, tests, distance within the building, regular ventilation, and hand hygiene – regardless of the severity of the pandemic situation outside the house, regardless of the duration of the pandemic) | yes/no | Hospital |

| **Personnel Management Indicators, Daily Collection** | **numerator** | **denominator** |
| --- | --- | --- |
| 1. Staff availability by qualification and ward/department | Actual available staff by qualification and ward/department | Potential staff (Number + FTE) by qualification and ward/department |
| 1. Number of employees by ward and qualification | Number of doctors, nursing staff, nursing assistants (+ FTEs) | Per ward |
| 1. Proportion of employees on vacation by ward and qualification | Number of employees on vacation (+ FTEs) by ward and qualification | Number of all employees per ward and qualification |
| 1. Total proportion of sick employees | Sick employees | All employees |
| 1. Absenteeism rate by qualification and ward | Number of sick employees (+ FTEs) by ward and qualification | Number of all employees (+ FTE) per ward and qualification |
| 1. Total proportion of staff not present at work on the current day | Employees do not present for duty | Number of all employees (+ FTE) |
| 1. Available nursing staff per patient | Available nursing staff | All patients |
| 1. Forecast of expected cases by ward (Normal, Intensive, requiring ventilation, ECMO) | Modeling results | - |
| 1. Forecast of expected workload on respective wards | Expected cases per ward | Daily workload per ward |

| **Quality and Patient Safety Monitoring (weekly, quarterly)** | **numerator** | **denominator** |
| --- | --- | --- |
| 1. Overall hospital mortality/by department | Deaths per week / in the department | All cases / per department per week |
| 1. Number of postponed indicated procedures / by service group | Diagnostic or therapeutic procedures delayed by at least one day | Scheduled diagnostic or therapeutic procedures per day and department and service group |
| 1. Number of unplanned readmissions (Ratio) | Number of unplanned readmissions per week | Number of discharges per week |
| 1. Number of inpatient-acquired fall-induced fractures | Number of patients with inpatient-acquired fall-induced fractures | Per 1000 patient days |
| 1. Number of inpatient-acquired pulmonary embolisms | Number of patients with inpatient-acquired pulmonary embolism | Per 1000 patient days |
| 1. Inpatient-acquired pressure ulcers | Patients with pressure ulcers of Grade/Category 2 to 4 or of unspecified grade/category, who were admitted without pressure ulcers or for whom it was not indicated that the pressure ulcer was present at the time of admission. | All patients treated as inpatients. |
| 1. Hospital mortality in live-born infants | In-hospital deaths of mature live-borns (>37 +0 to under 42+0 weeks) | All mature live-borns (>37 +0 to under 42+0 weeks) |
| 1. Decision-to-incision (D-I) time for emergency cesarean sections over 20 minutes | D-I-time > 20 min | All children born via emergency cesarean section. |
| 1. Cesarean section rates | number of cesarean births | number of births |
| 1. Tumor stage at first presentation of patients | defined according to the NRZ-KISS (national reference center for nosocomial infections – hospital information and surveillance system) | defined according to the NRZ-KISS |
| 1. Number of patients admitted with a cancer diagnosis per week | Distribution of tumor stages per entity on average in pandemic months | Distribution of tumor stages per entity among all patients first admitted with a cancer diagnosis (per entity) on average per month (non-pandemic months) |
| 1. Number of cancer patients admitted per week | Number of all patients first admitted with a cancer diagnosis (per entity) per week in pandemic months | Number of all patients first admitted with a cancer diagnosis (per entity) per week in non-pandemic months |
| 1. Number of cancer patients whose diagnostics or therapy is delayed due to hospital reasons | In cancer patients, diagnostic or therapeutic procedures delayed by at least one day | In cancer patients, scheduled diagnostic or therapeutic procedures per day and department |
| 1. Door-to-balloon time for initial PCI with ST-elevation myocardial infarction indication | Door-to-balloon time up to 60 minutes. | All initial PCIs (isolated PCI or simultaneous PCI) in patients with acute ST-elevation myocardial infarction at admission, in whom no fibrinolysis was performed prior to the procedure or this is unknown. Only procedures with valid information on the 'Door' and 'Balloon' timing, as well as with the date of the 'Door' and 'Balloon' timing from the year of data collection or the previous year, are considered. |
| 1. Time in the emergency department until decision on further treatment location/transfer | Average time in minutes that patients with a need for inpatient treatment wait in the emergency department until further treatment/transfer | All patients in the emergency department requiring inpatient treatment |
| 1. Number of overload reports per week | Overload reports per week/per 100 | All employees |
| 1. Number of suicides and suicide attempts during hospital stay (Psychiatric departments) | Number of suicides and suicide attempts by patients in psychiatric departments | Per 1000 patient days in psychiatric department |
| 1. Number of inpatient admissions with various mental illnesses per week (Psychiatric departments) | Number of inpatient admissions per psychiatric disease entity per week in pandemic weeks | Number of inpatient admissions per psychiatric disease entity per week in non-pandemic weeks (Psychiatric departments) |
| 1. Time to first imaging (Stroke) | Average time from inpatient admission to first imaging | All patients with suspected stroke |
| 1. Nosocomial, postoperative wound infection after inpatient surgeries (Non-implant surgeries) | defined according to the NRZ-KISS | All patients with non-implant surgeries |
| 1. Nosocomial, postoperative wound infections after inpatient surgeries (Implant surgeries) | defined according to the NRZ-KISS | All patients with implant surgeries |

| **Indicators for a Pandemic Dashboard; continuous monitoring during pandemics, regular practice of "in-line" data collection** | **numerator** | **denominator** |
| --- | --- | --- |
| 1. Regional pandemic incidence | New cases per day | Regional population (per 100,000) |
| 1. Hospitalization rate / hospital incidence | Hospital admissions of infected individuals per day | Infected per 100,000 |
| 1. Number of occupied beds by category, with staffing threshold in the hospital and department, separated by pandemic and other patients | Beds occupied with pandemic/non-pandemic patients per department | All beds for which staff is available by department |
| 1. Number of pandemic and other patients in the hospital | Number of pandemic patients // Number of non-pandemic patients | Hospital |
| 1. Number of admissions & discharges by wards into the hospital, separated by pandemic and other patients | Number of admissions of pandemic/non-pandemic patients // Number of discharges of pandemic/non-pandemic patients | All individual wards in the hospital |
| 1. Number of pandemic and other patients in general wards | Number of pandemic patients // Number of non-pandemic patients | All individual general wards |
| 1. Number of admissions & discharges in general wards, separated by pandemic and other patients | Number of admissions of pandemic/non-pandemic patients // Number of discharges of pandemic/non-pandemic patients | All individual general wards in the hospital |
| 1. Number of pandemic and other patients in intensive care units | Number of pandemic patients // Number of non-pandemic patients | All ICU wards in the hospital |
| 1. Number of admissions & discharges in intensive care units, separated by pandemic and other patients | Number of admissions of pandemic/non-pandemic patients // Number of discharges of pandemic/non-pandemic patients | All ICU wards in the hospital |
| 1. Proportion of intensive care patients infected with pandemic pathogens but not treated in the ICU because of the pathogen | All patients not in the ICU because of the pandemic pathogen | All patients in ICU infected with the pandemic pathogen |
| 1. Proportion of intensive care nursing staff, separated by pandemic and other patients | ICU nursing staff for pandemic patients / non-pandemic patients | All intensive care nursing staff |
| 1. Proportion of the total number of beds occupied by pandemic patients | Beds occupied by pandemic patients | All beds |
| 1. Proportion of pandemic and other patients / general ward beds | Pandemic patients / non-pandemic patients in general ward beds | All general ward beds |
| 1. Proportion of pandemic and other patients / intensive care beds | Pandemic patients / non-pandemic patients in ICU beds | All ICU beds |
| 1. Proportion of repurposed pandemic beds, occupied by pandemic patients | Repurposed beds occupied by pandemic patients | All beds repurposed due to the pandemic |
| 1. Proportion of repurposed intensive pandemic beds, occupied by pandemic patients | Repurposed ICU beds occupied by pandemic patients | All ICU beds repurposed due to the pandemic |
| 1. Proportion of occupied isolation beds, occupied by pandemic patients | Isolation beds occupied by pandemic patients | All occupied isolation beds |
| 1. Proportion of functional ventilators, separated by pandemic and other operations | Functional ventilators in wards available in the pandemic area / non-pandemic area | All ventilators on wards available in the pandemic area / non-pandemic area |
